# Correlation of the tumor escape phenotype with loss of PRELP expression in melanoma

**DOI:** 10.1186/s12967-023-04476-x

**Published:** 2023-09-20

**Authors:** Helene Schäfer, Karthikeyan Subbarayan, Chiara Massa, Christoforos Vaxevanis, Anja Mueller, Barbara Seliger

**Affiliations:** 1 Medical Faculty, Martin Luther University Halle-Wittenberg, Magdeburger Str. 2, 06112 Halle (Saale), Germany; 2 Fraunhofer Institute for Cell Therapy and Immunology, Perlickstr. 1, 04103 Leipzig, Germany; 3 Institute of Translational Medicine, Medical School Brandenburg, Hochstr. 29, 14770 Brandenburg an der Havel, Germany

**Keywords:** PRELP, Melanoma, MHC class I, Antigen processing, Immunogenicity, IFN signaling

## Abstract

**Background:**

Despite immunotherapies having revolutionized the treatment of advanced cutaneous melanoma, effective and durable responses were only reported in a few patients. A better understanding of the interaction of melanoma cells with the microenvironment, including extracellular matrix (ECM) components, might provide novel therapeutic options. Although the ECM has been linked to several hallmarks of cancer, little information is available regarding the expression and function of the ECM protein purine-arginine-rich and leucine-rich protein (PRELP) in cancer, including melanoma.

**Methods:**

The structural integrity, expression and function of PRELP, its correlation with the expression of immune modulatory molecules, immune cell infiltration and clinical parameters were determined using standard methods and/or bioinformatics.

**Results:**

Bioinformatics analysis revealed a heterogeneous, but statistically significant reduced PRELP expression in available datasets of skin cutaneous melanoma when compared to adjacent normal tissues, which was associated with reduced patients’ survival, low expression levels of components of the MHC class I antigen processing machinery (APM) and interferon (IFN)-γ signal transduction pathway, but increased expression of the transforming growth factor (TGF)-β isoform 1 (TFGB1) and TGF-β receptor 1 (TGFBR1). In addition, a high frequency of intra-tumoral T cells directly correlated with the expression of MHC class I and PRELP as well as the T cell attractant CCL5 in melanoma lesions. Marginal to low PRELP expression levels were found in the 47/49 human melanoma cell lines analysis. Transfection of PRELP into melanoma cell lines restored MHC class I surface expression due to transcriptional upregulation of major MHC class I APM and IFN-γ pathway components. In addition, PRELP overexpression is accompanied by high CCL5 secretion levels in cell supernatant, an impaired TGF-β signaling as well as a reduced cell proliferation, migration and invasion of melanoma cells.

**Conclusions:**

Our findings suggest that PRELP induces the expression of MHC class I and CCL5 in melanoma, which might be involved in an enhanced T cell recruitment and immunogenicity associated with an improved patients’ outcome. Therefore, PRELP might serve as a marker for predicting disease progression and its recovery could revert the tumorigenic phenotype, which represents a novel therapeutic option for melanoma.

**Supplementary Information:**

The online version contains supplementary material available at 10.1186/s12967-023-04476-x.

## Background

Melanoma, a malignant skin tumor, is estimated as the fifth most common cancer worldwide, with a steadily increasing incidence over the past decades. Although early-stage melanoma has a good prognosis after surgery, relatively small melanoma often exerts a high metastatic potential with a low 5 years survival rate of 15.7% before 2011 [1]. Due to its reported immunogenicity, different T cell-based immunotherapies have been developed for the treatment of melanoma. In particular, immune checkpoint inhibitors (ICPi) targeting the programmed cell death protein 1 (PD-1), PD-1 ligand (PD-L1) and/or the cytotoxic T lymphocyte antigen 4 (CTLA4) have significantly improved the outcome of patients with advanced melanoma [2, 3]. Since only a limited number of patients have a long-term response to ICPi, biomarkers for predicting response to immunotherapy and resistance development are urgently required. Recently, surface expression of HLA class I has been shown to serve as a predictive biomarker for ICPi response [4] or acquired resistance [5–7], suggesting that loss or downregulation of HLA class I expression might drive immune escape of melanoma. Indeed, Seliger and co-authors [8] provided one of the first evidences about structural alterations in components of the MHC class I antigen processing machinery (APM) and the interferon (IFN) signaling pathway in melanoma cells resulting in a loss of MHC class I surface expression. This could be reverted by gene transfer of APM and IFN components, which was associated with increased CD8^+^ T cell responses. Although genetic abnormalities are rare, the frequent downregulation of MHC class I expression in melanoma is mainly due to a deregulation of MHC class I APM molecules and/or molecules involved in IFN signaling at distinct levels [9–11]. It is noteworthy that a link between a low MHC class I surface antigens and low numbers of CD8^+^ T cells exists, which is associated with progression of melanoma [12, 13].

Since one primary goal in the field of tumor immunology is to increase the immunogenicity of tumor cells by re-establishing and maintaining MHC class I surface expression, the identification of key molecules or substances overriding these tumor intrinsic escape routes will help to improve durable elimination of cancer cells by CD8^+^ T cells.

In this context, proteoglycans (PGs) as crucial constituents of the extracellular matrix (ECM), which differ between tissues, developmental stages and (patho) physiological conditions, have been suggested to be involved in the immunogenicity of tumor cells by orchestrating cellular processes mediated by cell–cell and cell–matrix interactions [14] exhibiting tumor-suppressing and tumor-initiating properties [15–17]. Recently, a member of the small leucine-rich repeat proteoglycan (SLRP) family, named biglycan (BGN), has been shown as a regulator of MHC class I expression in HER‐2/neu- [18, 19] and K-RAS- [20] transformed cancer cells. However, little information exists about the role of other SLRPs on HLA class I expression, including the purine-arginine-rich and leucine-rich repeat protein (PRELP), also known as MST161, MSTp161, prolargin or SLRR2A [21]. PRELP consists of a core protein and interacts with collagen fibrils, heparin and heparan sulfate [22–27], proteins of the complement system, various membrane proteins [28, 29] as well as signaling pathways like the wnt and TGF-β [30]. The latter resulted in the suppression of Smad2 phosphorylation, leading to the inhibition of the TGF-β pathway [31]. PRELP is constitutively expressed in healthy cartilage, lung, kidney, liver and skin, secreted into the ECM [32] and involved in maintaining normal cellular structures and epithelial cell integrity. Next to its physiological function, PRELP has been reported as a biomarker in different human cancers, but its role is controversially discussed dependent on the tumor type analyzed [33–35] regarding its correlation with the patients’ survival [34, 36] and prognostic potential [30, 36–41]. While overexpression of PRELP has been reported to have tumor suppressive activity in solid tumors characterized by a reduced invasion, anchorage-independent growth, metastasis formation and tumorgenicity [42], PRELP expression is involved in the pathobiology of chronic lymphocytic leukemia [43]. Since no information exists for skin cancer, we investigated the expression, function and clinical relevance of PRELP in melanoma. Based on data from The Cancer Genome Atlas (TCGA), melanoma patients were divided into a PRELP^high^ and PRELP^low^ group and associated with clinical parameters, expression of HLA class I APM and IFN-γ signal pathway components, CCL5 as well as the tumor immune infiltration. These results were further confirmed in melanoma cell lines and the in vitro activity effect of PRELP on cell growth and immunogenicity was assessed.

## Methods

### Cell lines, treatment and transfection

Murine PRELP^low^ B16 F10 melanoma cells were purchased from the American Tissue Culture Collection. The different human melanoma cell lines were provided by S. Ferrone (Harvard Medical School, Boston, USA). Murine B16 F10 cells and the human melanoma cell lines were maintained in Eagles modified essential medium (EMEM, Lonza, Basel, Switzerland) supplemented with 10% fetal calf serum (FCS), 2 mM glutamine, 100 U/ml penicillin and 100 µg/ml streptomycin (PAA; Pasching, Austria) and cultured at 37 °C in 5% CO_2_ humified air.

PRELP-overexpressing (PRELP^high^) murine B16 F10 cells and human overexpressing cells (Buf 1088) as well as mock controls (PRELP^low/mock^) were generated as recently described for BGN [20]. Briefly, different melanoma cell lines were stably transfected with a PRELP expression vector according to Recktenwald and co-authors [44]. All stable PRELP transfectants and vector controls were maintained in complete EMEM supplemented with 1 mg/ml G418 (PAA).

### Determination of cell proliferation and migration

The growth properties of PRELP^low^ and PRELP^high^ melanoma cell lines were determined as recently described [45, 46]. Cell proliferation was analyzed after 48 h in triplicates using the cell proliferation kit II (Roche Applied Science, Penzberg, Germany) according to the manufacturers' instructions.

For determining cell migration, 5 × 10^4^ cells were plated into a trans-well chamber using a gradient of 0.5 to 10% FCS as an attractant in the lower chamber. After 24 h, the number of migrated cells into the lower chamber was determined using the Cell Titer Glo Luminescence cell viability assay (Promega) according to the manufacturer’s instructions. The luminescence was normalized against the luminescence cells that were directly seeded into the bottom of the trans-well plate. The results are expressed as % of migrated cells from at least three independent experiments using triplicates.

### RNA isolation, reverse transcription and quantitative real-time RT-PCR

Total cellular RNA was isolated from cell lines using the NucleoSpin RNA II kit (Macherey–Nagel, Dueren, Germany) followed by reverse transcription of 2 µg total RNA into cDNA and PCR as previously described [47] using target-specific primers listed in Additional file 1: Table S1. Experiments were independently repeated three times.

### Determination of the APM promoter activity by luciferase (luc) assays

TAP1, TAP2, TAPBP and PSMB9 promoter sequences were amplified from genomic DNA and then cloned into the pGl3 luciferase (luc) vector (Promega, Fitchburg, USA) as recently described [20, 48]. For transient transfections, 1 × 10^5^ cells were cultured in 100 µl OptiMEM (Invitrogen), followed by transfection with 0.3 µg promoter constructs and 0.016 µg β-galactosidase (β-gal) vector using Lipofectamine 2000 (Invitrogen, Waltham, USA) as transfection reagent according to the manufacturer’s instructions. 48 h after transfection, the luc activity was determined by adding the luc substrate (Promega) using a luminometer and normalized to the transfection efficiency determined by ß-gal enzyme activity. Experiments were independently done three times using triplicates.

### Western blot analysis

For Western blot analysis, 5 × 10^6^ cells were harvested, and proteins were extracted. 30 µg protein/lane was loaded in 10% SDS-PAGE gels, transferred onto nitrocellulose membranes (Schleicher & Schuell, Dassel, Germany) and stained with Ponceau S as previously described [47]. Membranes were incubated overnight at 4 °C with primary monoclonal antibodies (mAbs) directed against PRELP (Invitrogen, Waltham, USA), β-actin (Sigma-Aldrich, St. Louis, USA) and/or glyceraldehyde-3-phosphate dehydrogenase (GAPDH) (Cell Signaling Technology, Danvers, USA) as a loading control, respectively, followed by incubation for one hour with a horseradish peroxidase linked secondary antibody (Ab; Cell Signaling Technology) and developed using the ECL method. Chemiluminescence signals were visualized by the Lumi-Light Western Blotting Substrate (Roche Diagnostics) and recorded with a LAS3000 system (Fuji, Tokyo, Japan).

### Flow cytometry

For measuring MHC class I surface expression in murine and human melanoma cell lines, the following mAbs were used: the anti-H-2D mAb (Cedarlane Laboratories LTD, Burlington, Canada) and the anti-HLA class I-specific mAb (Beckman Coulter). Flow cytometric analysis was performed as previously described [48]. Briefly, 5 × 10^5^ cells were incubated with the appropriate amount of the respective antibodies at 4 °C for 30 min before the stained cells were measured on a NAVIOS (Becton Dickinson, Franklin Lakes, USA) and subsequently analyzed with the Kaluza Analysis Software (Beckman Coulter). The data are presented as mean specific fluorescence intensity (MFI) from three independent experiments.

For cell cycle analysis, 1 × 10^6^ cells were cultured in 0.1% FCS for 48 h followed by their cultivation in 10% FCS before cell cycle analysis [49]. Then, cells were fixed with ice-cold 70% ethanol and stained with propidium iodide (Sigma). The cells were subjected to flow cytometry using the Kaluza Analysis Software (Beckman Coulter). Three independent experiments were performed.

### Quantification of CCL5 by ELISA

For quantification of CCL5 in the supernatants of PRELP^low^ and PRELP^high^ melanoma cells, 2 × 10^4^ cells were seeded in 24 well plates. After an overnight incubation to allow cell adhesion, the medium was changed and cells were incubated for an additional 72 h. The supernatants were then harvested and the amount of CCL5 released by the melanoma cells was assessed by an ELISA (ELISA MAX™ Deluxe set, BioLegend) according to the manufacturer's instructions.

### CD107a degranulation assay

Peripheral blood mononuclear cells (PBMC) were purified from healthy human donors. Buffy coat was prepared by a gradient density centrifugation, then stimulated for 18 h with 1 ng/ml IL-12, 5 ng/ml IL-15 (Immunotools, Friesoythe, Germany) and 50 ng/ml IL-18 (Biovision, Milpitas, CA, USA) in X-vivo15 (Lonza) medium, before they were incubated together with PRELP^low^ vs. PRELP^high^ target cells. For the CD107a degranulation assay [50], the anti-CD107a Ab was added after 1 h of co-culture, while staining the cells with Abs directed against CD3, CD16 and CD56 to identify NK cells was performed after 4 h [19].

### Bioinformatics analyses of public datasets

The transcriptome profiles and clinical data from healthy individuals and cancer patients were obtained from the TCGA dataset (http://cancergenome.nih.gov/) and microarray data of the NCBI Gene Expression Omnibus (NCBI GEO) [51]. The datasets were available on GENT2 [52], HCMDB [53], UALCAN [54], R2 Genomics (http://r2.amc.nl), UCSC Xena (UCSC Xena (http://xena.ucsc.edu) platform and cBioPortal [55]. The number of human samples, cancers vs. adjuvant non-tumorigenic tissues and melanoma cell lines analyzed [56–60] are presented in Additional file 1: Table S2.

Using the Omic Horizon Expression database [61], PRELP expression was downloaded as transcripts per kilobase million (TPM) from multiple tissues and different sub-tissues according to the anatomical site. PRELP gene expression data were downloaded from the NCBI GEO database generated by the two microarray platforms Affymetrix U133A or U133Plus2. Then, the data were stratified into cancer and normal samples across 72 tissues by GENT2 system and relative PRELP expression was analyzed and correlated to cancer stage, age and clinicopathologic features. The comparison between cancer and normal tissues are presented as Log2FC (fold change calculates for logarithm to base 2) and p values are given.

PrognoScan (http://www.prognoscan.org/) was used to evaluate the relationship between gene expression and the survival of patients. Based on the gene expression levels, samples were divided into PRELP^high^ and PRELP^low^ expression groups and the differences in the risk and the overall survival (OS) between both groups (cut off 0.42) were analyzed and cumulative survival plots and Kaplan–Meier curves were constructed with the log-rank test. By using UCSC Xena, melanoma patients were categorized based on the global PRELP expression. The global PRELP expression ranges from log2 5.7 to 13. In the PRELP global range, PRELP^high^ refers to log2 < 10.6; the patients with log2 of < 8.1 were grouped as PRELP^low^. The Kaplan–Meier results are presented as log-rank test values and p values are given.

The OncoPrint algorithm in cBioPortal database [55] was used to analyze genetic alterations, such as amplification, deep deletions and mutations. A concise and compact graphical summary was generated for genomic alterations in PRELP across cutaneous melanoma samples. PRELP correlation analyses were performed with the R2: Genomics Analysis and Visualization Platform and correlation statistics were shown as Pearson correlation coefficients. The correlation of tumor immune cell infiltration and PRELP expression was analyzed as a co-variable to generate corrected multivariate Cox proportional hazard models. Results from the Cox regression models are presented as Hazard Ratios (HR) of z-scores and p values. In addition, the Tumor Immune Estimation Resource (TIMER) [62] was used to explore the associations of PRELP expression with the levels of immune cell infiltrates by employing the xCell algorithm [63]. The infiltration of CD8^+^ T cell subpopulations in tumor samples and PRELP were used for Cox univariate and multivariable regression analysis (p < 0.05), generating an independent Cox model.

### Statistical analysis

Microsoft Excel-Office 365 and R (programming language) were used for student’s t-test and one-way ANOVA. A p-value of < 0.05 was considered as statistically significant (*p < 0.05; **p < 0.01; ***p < 0.001). The survival curve was derived from the Kaplan–Meier method. The log-rank test was used to compare the survival rate.

## Results

### Reduced PRELP expression in melanoma

**Fig. 1:**
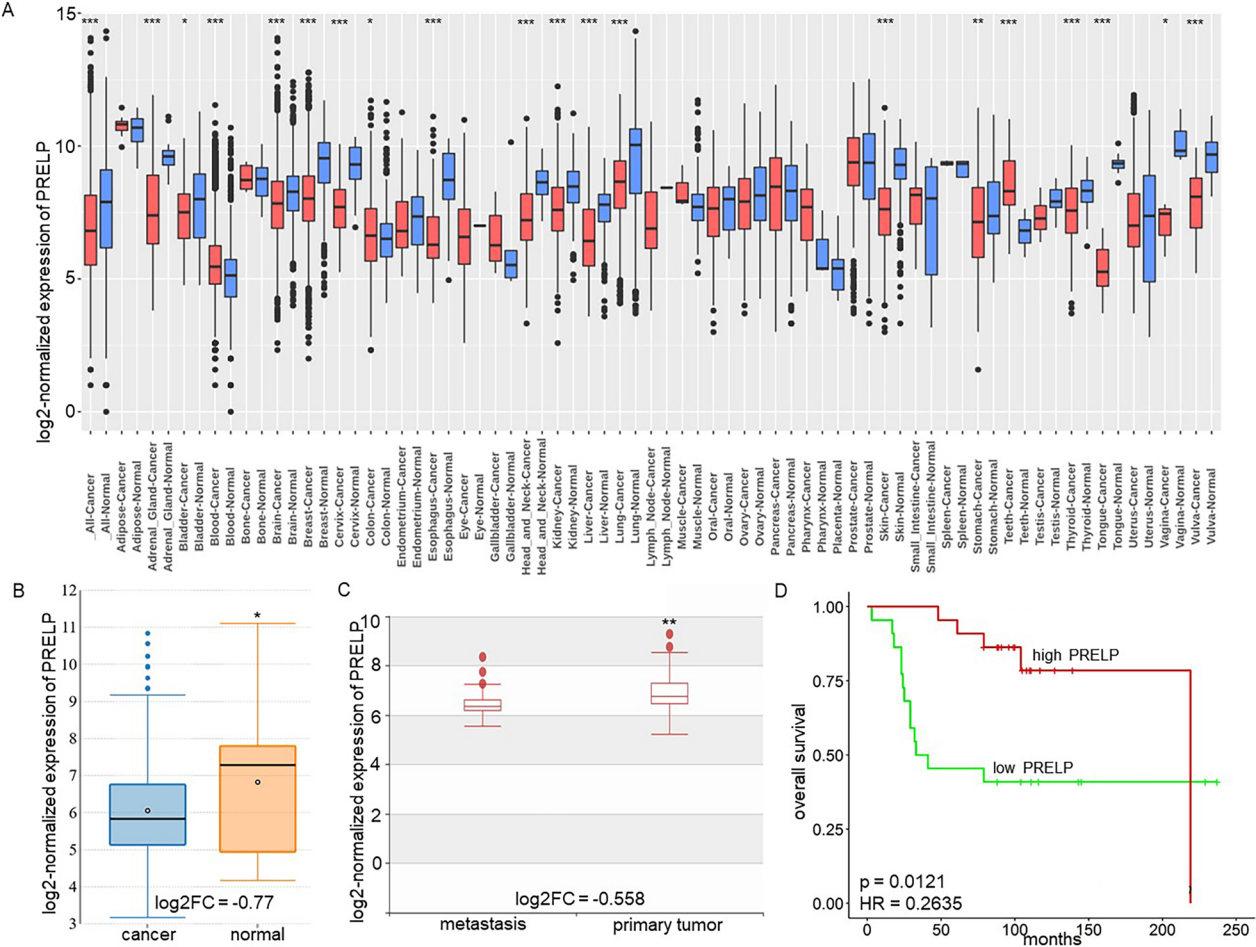
Correlation of PRELP expression in tumors of distinct origin and healthy controls and its clinical relevance. **A** Reduced PRELP expression in solid tumors compared to normal healthy tissues. PRELP expression was determined in 5.487 normal tissue samples and 35.806 cancer tissues. **B** Decreased PRELP expression in skin cancer cell lines compared to its normal counterparts. Box plots comparing PRELP expression in skin cancer (247 samples) vs. normal skin cell lines (32 samples) are shown. The data are expressed as log2FC-fold change in a logarithmic scale (base 2). **C** Distinct PRELP expression patterns in primary and metastatic skin tissues. A box plot comparing PRELP expression between metastases vs. primary tumors is shown. **D** Increased overall survival of melanoma patients with high PRELP expression levels in tumors. The Kaplan–Meier survival curve of PRELP^high^ and PRELP^low^ melanoma patients from the GEO (ID GSE98394; [68]) melanoma dataset was analyzed and presented as a Kaplan–Meier plot as described in “Methods”. The statistical significance is presented as *p < 0.05; **p < 0.01; ***p < 0.001

Members of the SLRP family exhibit pro-tumorigenic and anti-tumorigenic activities, dependent on the tissue, context, localization and tumor entity [14, 64–66], but limited information exists about their effects on the immunogenicity of tumor cells [26, 27]. A tissue-wide gene expression profile analysis of PRELP demonstrated a heterogeneous expression among 123 healthy human tissues and sub-tissues using the Omic Horizon Expression database. PRELP was highly expressed in cartilage (2963.97 TPM), aorta (1774.93 TPM), brain-superficial zone (1059.59 TPM), kidney-inner medulla (891.05 TPM) and Achilles tendon (840.84 TPM), whereas skin showed a moderate PRELP expression (110.45 TPM; Additional file 1: Fig. S1A). Since the expression of PRELP has only been analyzed in some tumor types, like bladder, pancreatic, colorectal, hepatocellular carcinoma and retinoblastoma [30, 36, 38, 41, 67], PRELP expression was determined in different cancers using the two independent databases GENT2–U133Plus2 and GENT2–U133A consisting of 5.487 normal tissues and 35.806 cancer samples. As shown in Fig. 1A, PRELP expression was generally downregulated in all solid cancers (log2FC = − 0.758 and p < 0.001), but there exist tumor-dependent differences in the extent of downregulation, which was more pronounced in melanoma (log2FC = − 1.583 and p < 0.001) when compared to corresponding normal tissues. However, PRELP is heterogeneously expressed in melanoma lesions leading to the assignment of PRELP^low^ and PRELP^high^ melanoma. These data are in line with cell line database analyses demonstrating downregulation of PRELP expression in skin cancer cells (log2FC = − 0.77 and p < 0.031) when compared to their normal counterparts, such as melanocytes and keratinocytes (GENT2—skin cancer cell lines) (Fig. 1B). In addition, 48 melanoma cell lines were tested for PRELP mRNA expression using qRT-PCR. Only 2/48 melanoma cell lines express high PRELP mRNA levels (Additional file 1: Fig. S1B), but the underlying mechanism of the lack of PRELP expression has not yet been determined.

### Clinical relevance of PRELP expression in melanoma

To get in-depth insights into the role of PRELP expression in cutaneous skin cancers, PRELP transcription was analyzed in an extensive series of primary and metastatic melanoma lesions and normal controls and compared to clinical parameters of melanoma patients (Tumor Skin Cutaneous Melanoma—TCGA, Additional file 1: Table S2). The PRELP mRNA expression levels were lower in primary melanoma lesions than in normal skin (log2FC = − 1.245 and p = 2.485e−3) and further reduced in metastasis (log2FC = − 0.558 and p = 3.047e−2) (Fig. 1C), but independent of nodal metastasis status, disease stage, patient’s age and gender (Additional file 1: Fig. S1C; GEO ID: GSE7553) [69]. Kaplan–Meier analysis revealed that PRELP expression levels correlated with the overall survival (OS) of melanoma patients with a reduced patients’ survival rate (HR = − 0.2635; p = 0.0121) in PRELP^low^ melanoma lesions (Fig. 1D).

### Link of PRELP expression with the immune escape phenotype

**Fig. 2:**
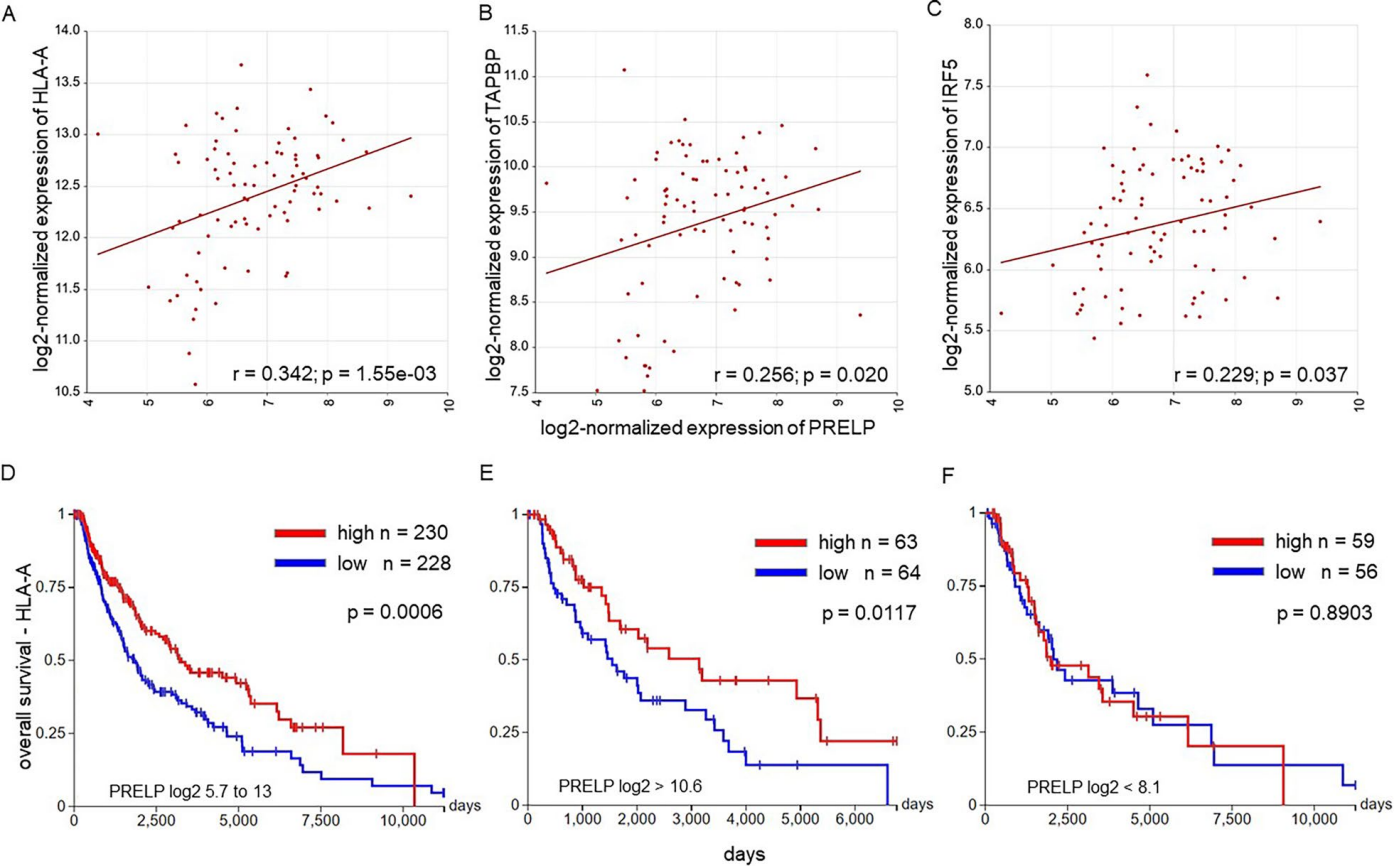
Correlation of PRELP expression with HLA class I expression. **A**–**C** Correlation of PRELP expression with HLA-A, TAPBP and IRF5. Using the GEO (ID GSE8401) melanoma dataset and R2 Genomics for PRELP expression was correlated to HLA-A (**A**), TAPBP (**B**) and IRF5 (**C**) mRNA levels. **D**–**F** Correlation of PRELP^high^ vs. PRELP^low^ melanoma with the expression of HLA-A and overall survival. Data of Tumor Skin Cutaneous Melanoma—TCGA were analyzed and results are depicted as Kaplan–Meier plots. Correlation of the OS for HLA-A expression independent of PRELP expression (**D**), PRELP^high^ (**E**) and PRELP^low^ (**F**) samples are shown in Kaplan–Meier plots

In order to determine whether PRELP expression is inversely associated with an immune escape phenotype, the HLA class I expression was analyzed in PRELP^high^ vs. PRELP^low^ melanoma lesions by in silico analysis using three different human melanoma data sets: (i) mixed melanoma metastasis (83 samples; GEO ID: GSE8401) and (ii) tumor skin cutaneous melanoma (480 samples; TCGA) (Additional file 1: Table S3). A statistically significant positive correlation to PRELP was found for HLA class I antigens (Fig. 2A) and TAPBP (Fig. 2B) in two datasets for at least one proteasome subunit (Additional file 1: Table S3). By comparison of the HLA-A expression in 133 skin cancer cell lines (GENT2—skin cancer cell lines) with PRELP, a significant positive correlation, Pearson correlation coefficient, r = 0.37 and p = 1.98E−09 (Additional file 1: Fig. S2A). In addition, a positive correlation was also detected between PRELP and IRF5 expression (Fig. 2C). The data from melanoma lesions were in line with a strong downregulation of MHC class I surface expression in murine and human melanoma cells, which exhibit low to marginal PRELP expression (PRELP^low/neg^) in 46/48 melanoma cell lines analyzed. Thus, a link between high PRELP expression levels and increased immunogenicity of melanoma cells is shown in vitro and in vivo.

Further analysis of the Tumor Skin Cutaneous Melanoma—TCGA dataset revealed that a high HLA-A expression level favored an increased OS of melanoma patients (p = 0.0006; log-rank test = 11.76) regardless of PRELP expression levels (log2 5.7 to 13) (Fig. 2D), but PRELP^high^ expression (log2 > 10.6) was accompanied by high levels of HLA class I expression and increased patients’ OS (p = 0.01168; log-rank test = 6.359) (Fig. 2E). In contrast, in PRELP^low^ melanoma (log2 < 8.1), HLA-A failed to predict the survival of melanoma patients (p = 0.8903; log-rank test = 0.019; Fig. 2F).

### Reversion of the tumorigenic phenotype by PRELP overexpression in melanoma cell lines

**Fig. 3:**
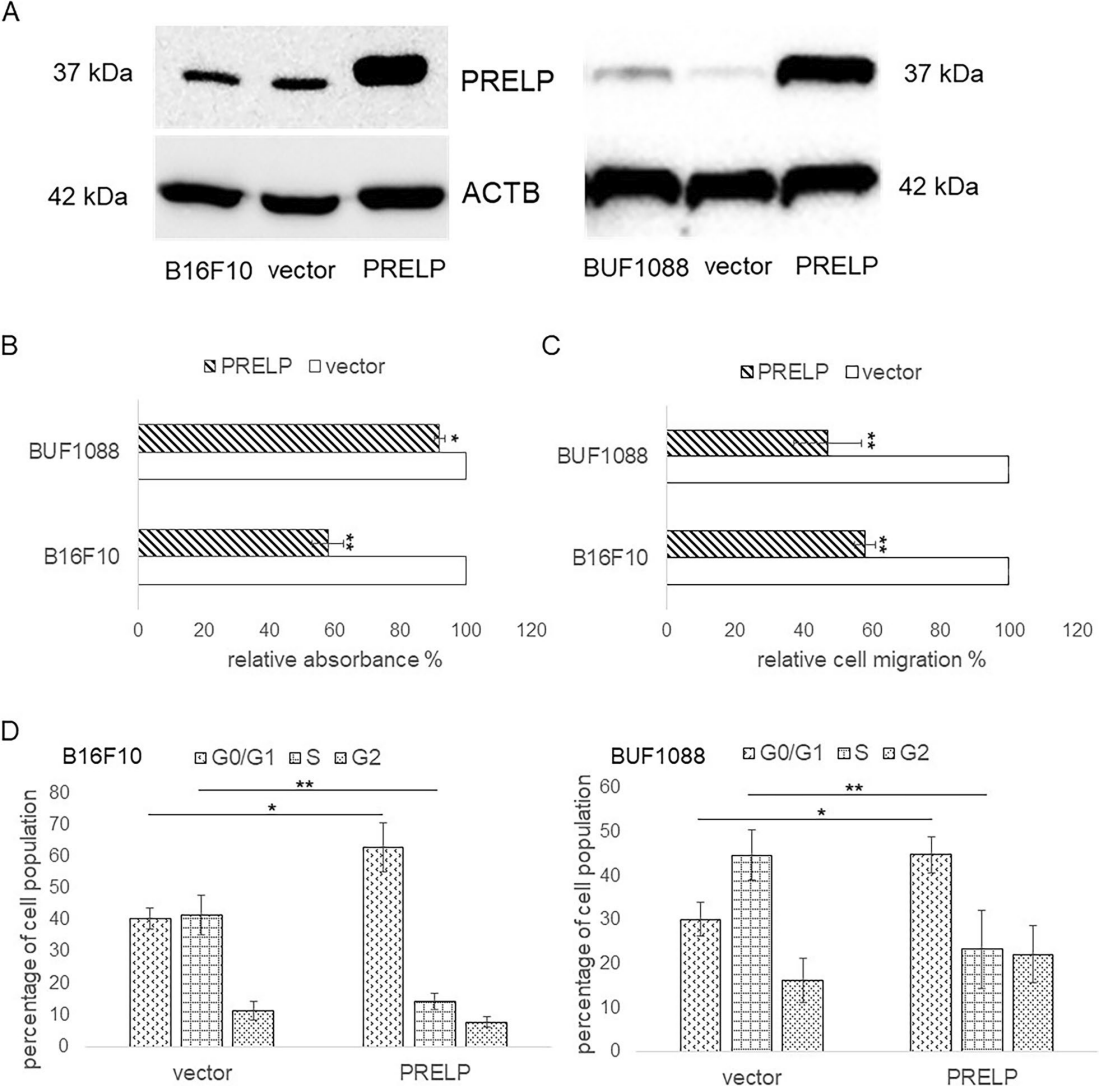
Restoration of PRELP expression in melanoma cells and its effect on growth properties. Murine and human PRELP^low^ melanoma cells by transfection. Restoration of PRELP expression. **A** PRELP expression in PRELP^high^ vs. PRELP^low^ expressing melanoma cells was determined by Western blot analysis as described in “Methods”. A representative WB using an anti-PRELP Ab is shown and the PRELP protein expression (37 kD) is marked. **B** Reduced proliferation of PRELP^high^ vs. PRELP^low^ expressing melanoma cells. The growth properties of PRELP^high^ vs. PRELP^low^ B16F10 and Buf1088 cells were determined as described in “Methods”. The proliferation rates of PRELP^low^ and PRELP^high^ melanoma cells were correlated to that of PRELP^low^ vector controls, which were set to “100”. The data are represented in bar charts as the mean of three independent experiments. **C** Altered migration capacity of PRELP^low^ vs. PRELP^high^ melanoma cells. The migration rate of PRELP^low^ vs. PRELP^high^ B16F10 and Buf1088 cells was determined by ATP-based fluorescence as described in “Methods” and normalized to the seeding control. The graph represents the % of migrated PRELP^low^ and PRELP^high^ melanoma cells as a mean of three independent experiments. **D** Altered cell cycle in PRELP^low^ vs. PRELP^high^ melanoma cells. Cell cycle distribution of PRELP^low^ vs. PRELP^high^ B16F10 and Buf1088 was assessed by flow cytometry as described in “Methods”. The data were presented as the % of cells in the different cell cycle phases as the mean of three independent experiments demonstrating a cell cycle arrest in the G0/G1 phase of PRELP^high^ melanoma cells. The statistical significance is presented as *p < 0.05; **p < 0.01; ***p < 0.001

Transfection of PRELP^low/neg^ B16F10 and Buf1088 cells with a PRELP expression vector restored PRELP mRNA (Additional file 1: Fig. S3) and protein expression (Fig. 3A). When compared to PRELP^low/neg^ B16F10 and Buf1088 cells PRELP overexpressing (PRELP^high^) B16F10 and Buf1088 transfectants exhibited altered growth properties, such as reduced cell proliferation (Fig. 3B) and reduced migration determined by trans-well invasion assay (Fig. 3C) accompanied by a cell cycle arrest in the G0/G1 phase (Fig. 3D).

**Fig. 4:**
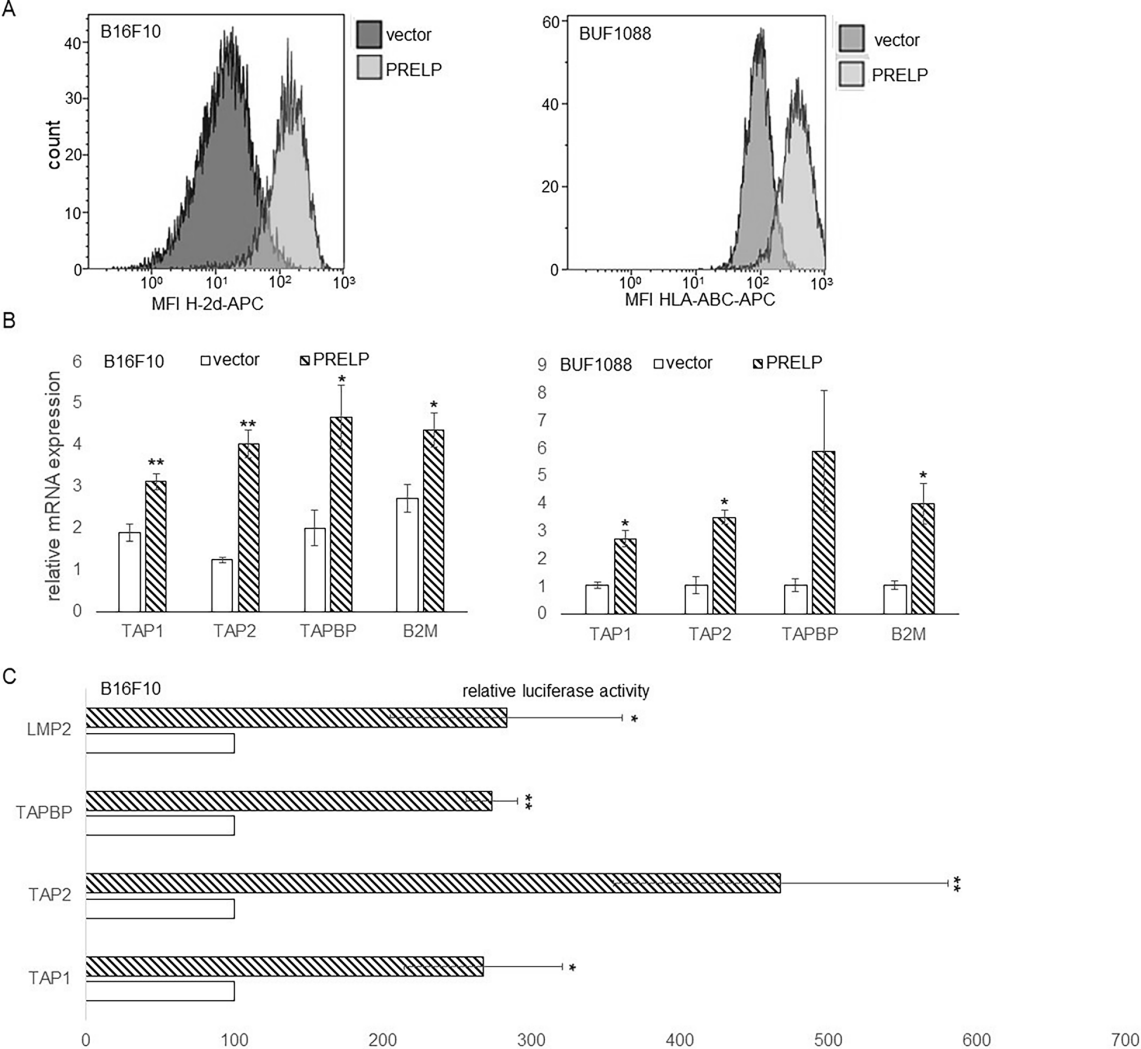
Upregulation of MHC class I APM and IFN signaling components and altered NK cell response by restoration of PRELP in PRELP^low/neg^ B16F10 and Buf1088 cells. **A** PRELP-mediated upregulation of MHC class I surface antigen expression. MHC class I surface expression was assessed by flow cytometry in PRELP^low/neg^ vs. PRELP^high^ B16F10 and Buf1088 cells as described in “Methods”. The data are represented as histograms of MHC class I surface expression of PRELP^low/neg^ vs. PRELP^high^ B16F10 and Buf1088 cells. **B** PRELP-mediated upregulation of MHC class I APM components expression in melanoma cells. The mRNA expression levels of MHC class I APM components in PRELP^low/neg^ vs. PRELP^high^ melanoma cells were analyzed by qPCR as described in “Methods”. The results are expressed in bar charts representing the mean of three independent experiments as relative mRNA expression levels of selected APM components in PRELP^low/neg^ and PRELP^high^ B16F10 and Buf1088 cells. **C**. Transcriptional downregulation of selected MHC class I APM components in PRELP^low/neg^ melanoma cells. APM promoter activity in PRELP^low/high^, mock transfectants and two independent PRELP transfectants of B16F10 cells was determined by luciferase assays as described in “Methods”. The data were normalized to *β*-gal activity and presented in a bar chart as the mean of the relative luc activity of at least three independent experiments. Error bars indicate the standard error. The statistical significance is presented as *p < 0.05; **p < 0.01

Furthermore, PRELP^high^ B16F10 and human Buf1088 cells showed a strong upregulation of MHC class I surface expression (Fig. 4A), which was accompanied by an increased expression of major components of the MHC class I APM, like the transporter-associated with antigen processing (TAP)1 and TAP2, tapasin (TAPBP), β_2_-microglobulin (β_2_-m) and IFN-γ-induced proteasome subunits (Fig. 4B).

**Fig. 5:**
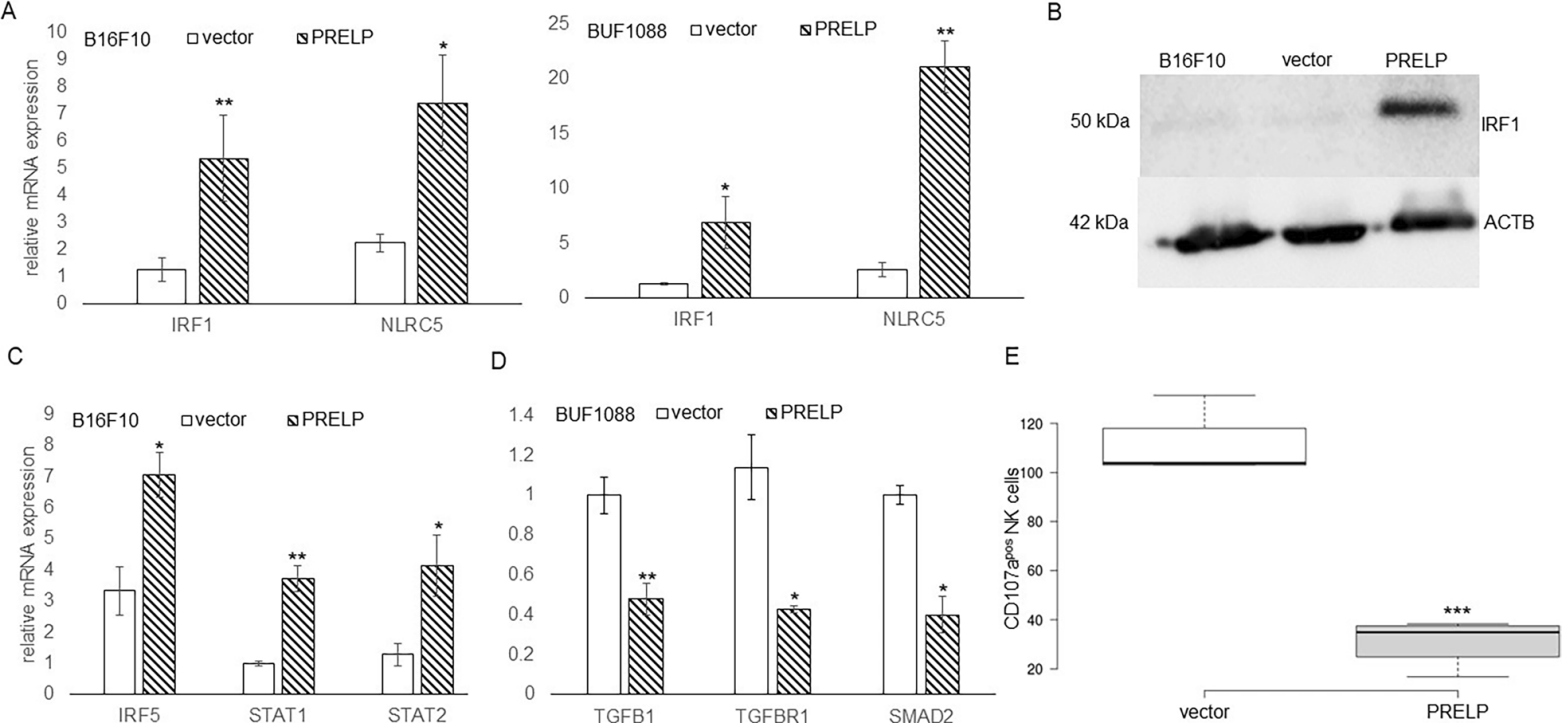
Altered expression of IFN-γ signal components, NLRC5 and TGF-β pathway molecules in PRELP^high^ vs. PRELP^low/neg^ melanoma cells. **A** Increased mRNA levels of IRF1 and NLRC5 in PRELP^high^ vs. PRELP^low^ melanoma cells were assessed by qPCR as described in “Methods” for IRF1 and NLRC5 expression. The qPCR data are expressed in bar charts relative to parental cells (set 1) and represent the mean ± SE format from three independent experiments. **B** A representative WB using an anti-IRF1 Ab is shown and the PRELP protein expression is marked. **C** The mRNA levels of IRF5, STAT1 and STAT2 were determined by qPCR as described in “Methods”. The qPCR data are shown in bar charts relative to parental cells (set 1) and represent the mean ± SE format from three independent experiments. **D** Altered TGF-β signaling components in PRELP^high^ B16F10 cells. The expression levels of TGFB1, TRFBR1 and SMAD2 were determined by qPCR. The data are expressed in bar charts relative to parental cells (set 1) and represent the mean ± SE format from three independent experiments. **E** Reduced NK cell activity in PRELP^high^ vs. PRELP^low^ Buf1088 cells. CD107a degranulation assay was performed as described in “Methods” by co-culture with NK cells from three different donors with PRELP^low^ vs. PRELP^high^ Buf1088 cells. The mean ± SE of the CD107a degranulation of PRELP^low^ vs. PRELP^high^ Buf1088 cells using NK cells representing total NK cell activity are shown. The statistical significance is presented as *p < 0.05; **p < 0.01; ***p < 0.001

Since the components of the IFN-γ signal transduction and NLRC5 have been linked to MHC class I APM component expression [9, 70], these molecules were also analyzed in PRELP^low^ vs. PRELP^high^ murine and human melanoma cell lines cells. PRELP^high^ melanoma cells express increased levels of NLRC5 (Fig. 5A) as well as selected IFN-γ signal transduction molecules, e.g., IRF1 (Fig. 5B), IRF5, STAT1 and STAT2 (Fig. 5C).

Based on the known effect of PRELP on the TGF-β signaling, a possible link between PRELP and MHC class I expression with this pathway was also investigated. As expected, the TGF-β receptor (TGFBR1), its ligand (TGF-β1) and SMAD2 were downregulated in PRELP^high^ Buf1088 cells (Fig. 5D). The functional impact of the PRELP-mediated MHC class I upregulation on NK cell responses was analyzed in Buf1088 cells using a CD107 degranulation assay. As shown in Fig. 5E, a reduced NK cell recognition of PRELP^high^ compared to PRELP^low^ Buf1088 cells was found.

### Underlying molecular mechanisms of low PRELP expression and PRELP-mediated downregulation of MHC class I in melanoma

To understand the molecular mechanisms of low PRELP expression in melanoma, the frequency of structural alterations in the PRELP gene was determined in 287 melanoma samples of the TCGA dataset (Tumor Skin Cutaneous Melanoma—TCGA) with available mutation and copy number alteration data. As shown in Additional file 1: Fig. S4, mutations only occurred in 20/287 melanoma samples (7%) with 7 missense mutations with unknown significance distributed over the whole gene. Based on these bioinformatics data, it was hypothesized that the low PRELP expression levels in melanoma might be mainly due to deregulation rather than genomic abnormalities.

To determine the PRELP-mediated downregulation of MHC class I APM component expression, the promoter activity of selected APM components was analyzed by luciferase (luc) reporter assays in PRELP^low^ vs. PRELP^high^ melanoma cells. Transient transfection of the APM promoter luc constructs and vector controls in parental PRELP^low/neg^ B16F10 cells and PRELP^high^ B16F10 transfectants demonstrated a strong induction of luc activity in PRELP^high^ compared to PRELP^low/neg^ B16F10 cells, but with differences in the promoter activity levels depending on the APM component analyzed (Fig. 4C).

### Link of PRELP expression with immune cell infiltration and CCL5 in melanoma cell lines

**Fig. 6:**
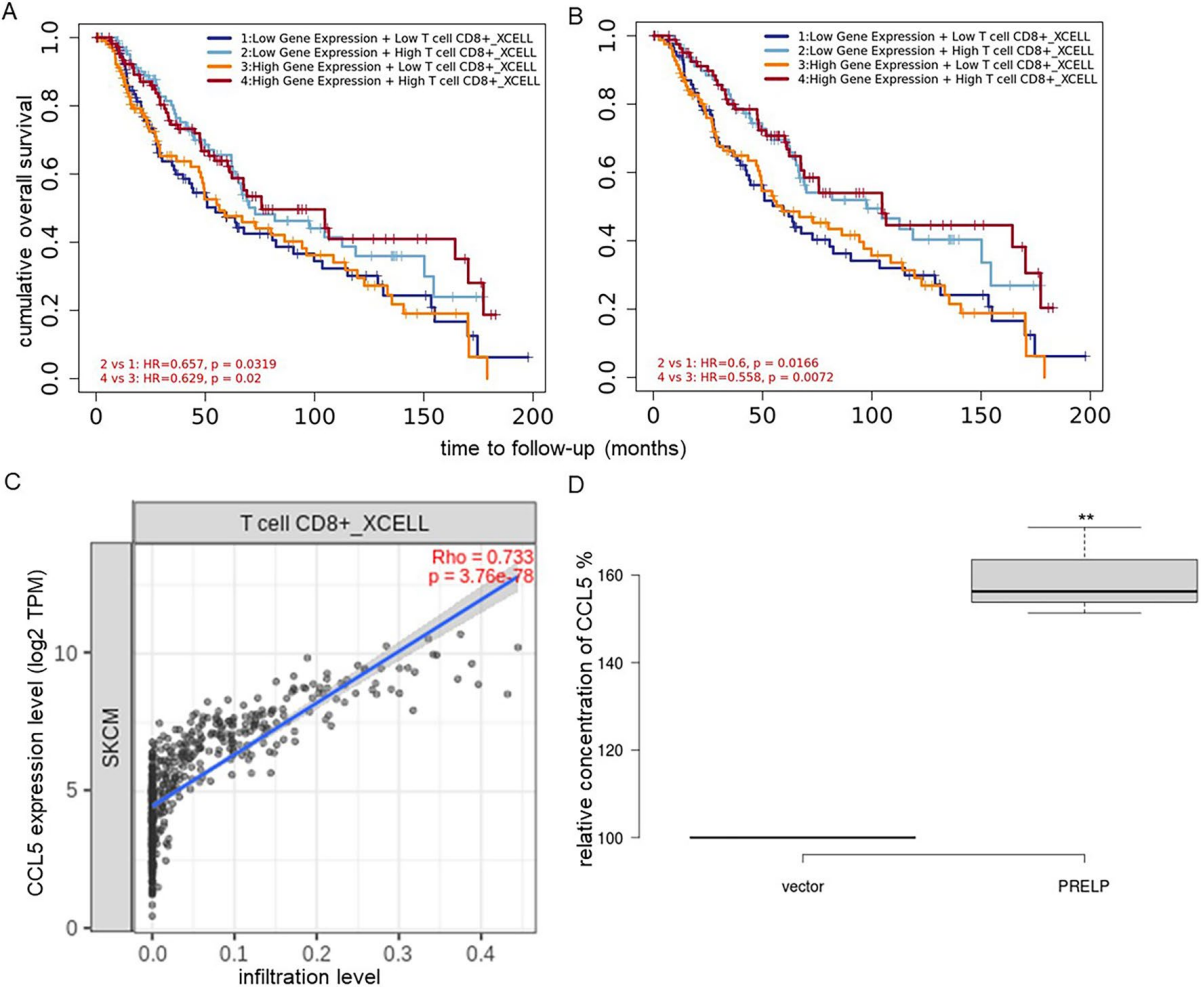
Link of CD8^+^ T cell infiltration levels with PRELP and CCL5 expression in melanoma. **A**, **B** Data sets of tumor skin cutaneous melanoma (**A**) and metastatic melanoma (**B**) were analyzed by bioinformatics and linked to immune cell infiltration. The prognostic signature of the infiltration of CD8^+^ T cell subpopulations and the expression of PRELP profiled by xCell algorithm is represented as the Cox model. **C** A correlation plot was displayed between CCL5 and CD8^+^ T infiltration in a cohort of 480 melanoma samples of the TCGA dataset. **D** Secretion of CCL5 in PRELP^high^ vs. PRELP^low^ melanoma cells. Supernatants of PRELP^high^ and PRELP^low/negative^ Buf1088 cells were analyzed for the production of the chemokine CCL5 cells using ELISA as described in “Methods”. Representative data from three independent experiments are shown. The statistical significance is presented as **p < 0.01

In order to determine whether there is a link between high PRELP expressions associated with high HLA class I levels and immune cell infiltration, immune cell infiltration was determined in correlation to PRELP expression. As shown in Fig. 6A, high expression of PRELP in tumors correlated with a high number of tumor-infiltrating CD8^+^ T cells in skin cutaneous (HR = − 4.079, p = 1.25e−04) and metastatic melanoma (HR = − 3.758; p = 6.19e−04) (Fig. 6B).

In-depth analysis of T cell subpopulations revealed that high PRELP expression levels in melanoma were significantly correlated to high infiltrations of naive (HR = − 2.810; p = 1.36e−02 and HR = − 2.805; p = 1.72e−02), central memory (HR = − 4.326; p = 4.54e−05 and HR = − 3.973; p = 2.71e−04) and effector memory (HR = − 2.807; p = 1.25e−02 and HR = − 2.676; p = 2.3e−02) CD8^+^ T cells in both tumor skin cutaneous (Additional file 1: Fig. S5A) and metastatic melanoma (Additional file 1: Fig. S5B) datasets.

Comparable to tumor-infiltrating CD8^+^ T cells, high PRELP expression in SKCM was correlated with an increased number of tumor-infiltrating monocytes (HR = − 3.077; p = 5.87E-03 and HR = − 2.832; p = 1.59E−02), macrophages (HR = − 3.934; p = 3.01E−04 and HR = -3.46; p = 2.23E−03), myeloid dendritic cells (HR = − 2.619; p = 2.30E−02 and HR = − 2.375; p = 5.47E−02) in both skin cutaneous and metastatic melanoma datasets (Additional file 1: Table S4). The number of tumor-infiltrating B cells and tumor PRELP expression were associated with a decreased risk of skin cutaneous melanoma (HR = − 2.533; p = 2.81E−02), which was not significant for metastatic melanoma (HR = − 1.995; p = 1.25E−01). In contrast to the associations described, a high frequency of common myeloid progenitors was associated with an increased risk (HR = 2.284; p = 4.84E−02) in PRELP^high^ skin cutaneous melanoma patients. The frequency of other infiltrating cell subpopulations listed in Additional file 1: Table S4, including CD4^+^ T cell subpopulations, innate immune cells, cells of the hematopoietic lineage and stroma cells, lack significant clinical relevance. Since the chemokine CCL5 is known to recruit CD8^+^ T lymphocytes to the site of inflammation [71], the frequency of CD8^+^ T infiltration in tumors was correlated with the CCL5 expression in a cohort of 480 melanoma samples of the TCGA dataset. A strong positive correlation (Rho = 0.733; p = 3.76e−78) between CCL5 and CD8^+^ T infiltration was found (Fig. 6C). Based on these data, the effect of PRELP overexpression on CCL5 secretion in Buf1088 melanoma cells was determined using the ELISA. When compared to PRELP^low^ parental cells, PRELP^high^ Buf1088 showed higher secretion levels of CCL5 into the cell supernatant (Fig. 6D). This is consistent with the TCGA data of global PRELP (log2 5.7 to 13) (Additional file 1: Fig. S6A) and PRELP^high^ (log2 > 10.6) (Additional file 1: Fig. S6B) melanoma with a higher OS (global PRELP; p = 0.00006; log-rank test = 17.72; PRELP^high^; p = 0.002; log-rank test = 9.472) upon high CCL5 expression. In contrast, CCL5 has no prognostic relevance in PRELP^low^ patients (p = 0.163; log-rank test = 1.950; log2 < 8.1) (Additional file 1: Fig. S6C).

## Discussion

PRELP has been shown to affect the immunogenicity of tumors by upregulating MHC class I surface expression and activating IFN signaling, which could modulate tumor development as well as the TME leading to an enhanced CD8^+^ T cell infiltration. Based on the activity of PRELP on tumor cell function described in this study, this SLRP is suggested as a candidate for anti-cancer therapy. However, so far there exist only a few experimental studies that consider the therapeutic role of this molecule. Since upregulation of PRELP has been demonstrated to be associated with anti-tumoral activity, PRELP should be reconstituted in melanoma, which might be a novel form of anti-cancer strategy. In addition, compounds should be identified, which upregulate PRELP expression leading to anti-cancer activity. Next to its therapeutic potential, our bioinformatics pan-cancer analysis as well as our functional results demonstrated the potential use of PRELP as prognostic and therapeutic markers. Due to a dual role of some SLRPs in cancer, further characterisation of key ECM molecules in tumor progression or tumor suppression is required [72–74].

During the last decade, increased information exists about the role and function of ECM components in tumors, which are altered at the biomechanical, biochemical, architectural and topographic level [14]. The ECM has been further shown to be an essential and dynamic part of the TME [75] and undergoes remodeling mediated by several matrix-degrading enzymes during normal and pathologic conditions. A deregulation of the ECM composition and structure has been associated with the development of tumor progression by a detachment of tumor cells from each other, from adjacent immune and stromal cells and contributes to increased cell proliferation, migration and invasion as well as reduced patients’ survival [76]. In addition, ECM components affect the EMT and dissemination of cells, the composition of the TME and tumor immunogenicity [76, 77].

In general, SLRPs were first correlated with the regulation of innate immune responses, thereby generating a pro-inflammatory TME, which could trigger tumorigenesis [78]. However, some SLRPs, an oncogenic and/or tumor-suppressive role has been discussed. For example, a link between the BGN-mediated downregulation, oncogenic transformation and a reduced immunogenicity due to low MHC class I surface expression levels was reported. This was caused by transcriptional suppression of major APM components and an increased TGF-β signaling [18]. In addition, the SLRP decorin also exhibited anti-tumorigenic properties by downregulating the release of IL-10 thereby inhibiting tumor growth [79]. It can also alter the tumor stroma and modulate the tumor-associated inflammation [80].

**Fig. 7:**
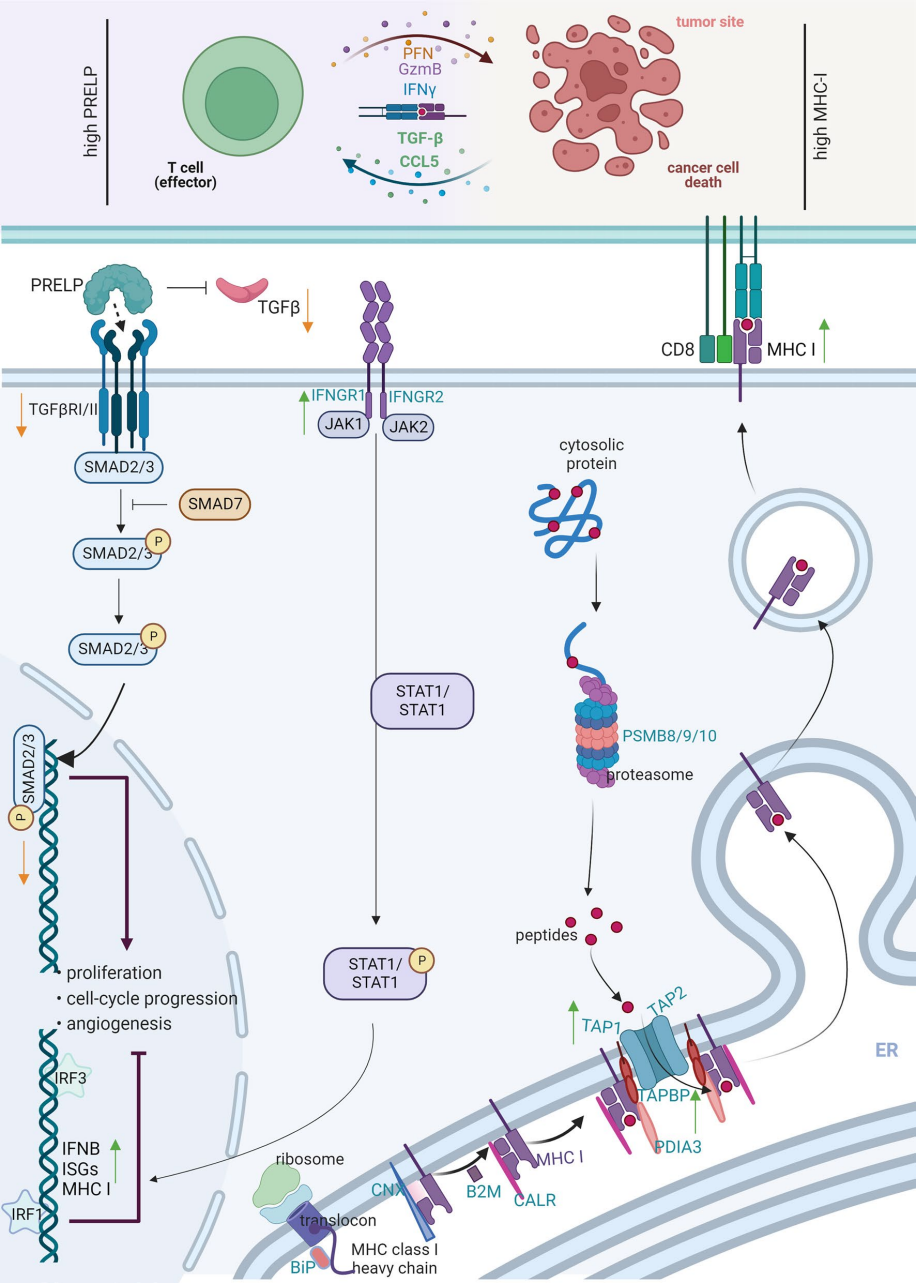
Schematic diagram of PRELP activity in hallmarks of cancer. The reduced PRELP expression in melanoma cells is associated with low expression levels of MHC class I APM components and members of the IFN-γ signal transduction as well as CCL5, which could be reverted by reconstitution of PRELP. PRELP induces the expression of MHC class I and secretion of CCL5 in melanoma cells, which contributes to the recruitment of effector T cells into the tumor tissues and increased recognition by CD8^+^ T cells (created with BioRender.com)

Next to BGN, the expression of PRELP was also downregulated in K-RAS- and/or HER-2/neu-transformed fibroblasts [44]. Based on these results, a tumor-suppressive activity of PRELP in tumor cells has been suggested. Indeed, our results demonstrated a PRELP-mediated induction of the expression of MHC class I and secretion of CCL5 in melanoma cells (Fig. 7), which might contribute (i) to the recruitment of effector T cells into the tumor tissues and (ii) to an increased recognition by CD8^+^ T cells.

This is in line with recent reports demonstrating a tumor inhibitory capacity of PRELP [38, 40], which might be at least partially due to its ability to directly bind TGFB1 [30, 31, 34]. Interestingly, our study demonstrated a reduced expression of TGFB1, TFGBR1 and SMAD2 in PRELP^high^ vs PRELP^low^ melanoma cells.

Furthermore, bioinformatics analyses of high throughput TCGA and GEO data showed a reduced PRELP expression occurring at a high frequency in solid cancers compared to adjacent normal tissues, including primary melanoma lesions, which was even more pronounced in melanoma metastases. Furthermore, low levels of PRELP expression were associated with a low T cell infiltration and low HLA class I expression suggesting that an impaired PRELP expression of tumor cells represents an immune escape phenotype.

This is further in line with a reduced expression of CCL5 in PRELP^low^ melanoma, which is a potent chemoattractant for T cells. CCL5 plays an important role in recruitment of effector T cells to the tumor site. Furthermore, its expression shows a positive correlation with both increased T cell infiltration and the survival of patients with cancer [81]. Indeed, a positive correlation exists between increased numbers of tumor-infiltrating monocytes and CD8^+^ T cells and the expression of CCL5 in melanoma, but also in other types of cancer, which further helped the recruitment of CD8^+^ effector T cells [71, 82, 83]. Thus, the expression of PRELP appears to be linked with the composition of the TME and the cellular immune responses.

Concerning the underlying molecular mechanisms leading to PRELP downregulation, genomic abnormalities in PRELP, such as mutations and deletions, were only found at a low frequency (7%) suggesting deregulation as a major mechanism of impaired PRELP expression in melanoma (Additional file 1: Fig. S3). This could occur at the epigenetic, transcriptional as well as post-transcriptional levels. However, the underlying mechanisms of PRELP expression in 2/48 melanoma cell lines have not been identified. Restoring PRELP expression might have anti-tumoral activity and could be combined with immunotherapies to enhance treatment efficacy.

There exists increasing evidence about the clinical relevance of PRELP as a tumor suppressor in solid tumors and its use as a prognostic marker [30, 38, 84]. In this study, the PRELP expression status was significantly associated with the patients’ prognosis. Melanoma patients with low PRELP expression had a shorter OS, which was not associated with the metastatic status, age and gender. These data were confirmed by in vitro analysis of human and murine melanoma cell lines and were in line with downregulation of PRELP in CRC specimens compared to adjacent normal mucosa [41].

Since proliferation, invasion and metastasis formation are significant features of melanoma, the effect of PRELP overexpression on growth properties was analyzed, which might also lead to identifying novel therapeutic targets controlling disease progression. In accordance with recently published results for hepatocellular carcinoma [38], PRELP overexpression inhibited cell proliferation, migration and invasion of murine and human melanoma cell lines. However, the inhibitory role of PRELP on melanoma growth has to be investigated in more detail and might be associated with the modulation of multiple signal transduction pathways, such as β-catenin or NF-_K_B signaling [85, 86]. PRELP treatment has been shown to suppress cancer progression by inhibiting the TGF-β and EGF pathways leading to the control of the EMT [30]. Interestingly, an altered TGF-β signaling was also shown in BGN-overexpressing cells, which was associated with an increased SMAD2 expression due to the inhibition of miR-21. This results in an upregulation of MHC class I APM components and an increased immunogenicity of BGN^high^ vs. BGN^low^ cells.

In sum, this study extends the knowledge about the role of SLRPs regarding their tumor suppressive activity [19]. Not only BGN, but also PRELP has a positive immune modulatory potential by increasing the expression of HLA class I APM and IFN-γ signaling components suggesting a tumor suppressive activity of PRELP in melanoma. Vice versa, reduced PRELP expression in melanoma cells is associated with low expression levels of MHC class I APM components and members of the IFN-γ signal transduction as well as CCL5, which could be reconstituted by PRELP overexpression (Fig. 7). Since genetic alterations were not identified, the impaired PRELP expression in melanoma might be mainly due to its deregulation rather than structural abnormalities. In addition, high PRELP expression is significantly correlated with increased patients’ survival and CD8^+^ T cell infiltration, which might be associated with enhanced T cell responses in melanoma. Therefore, PRELP is a prognostic biomarker and might be a potential (immune) therapeutics for enhancing CD8^+^ T cell responses, thereby leading to novel approaches for the treatment of melanoma patients.

### Supplementary Information

**Additional file 1: Figure S1.** PRELP expression in neoplastic and non-neoplastic human tissues and cells. **Figure S2.** Correlation plot of HLA-A and PRELP in 133 skin cancer cell lines (GENT2—skin cancer cell lines). **Figure S3.** Reconstitution of PRELP expression in melanoma cells. Overexpression of PRELP in melanoma cell lines was obtained after transfection of a PRELP expression vector in murine and human PRELP^low^ melanoma cells. Transfection with a mock vector served as control. **Figure S4.** Distribution of gene mutations of PRELP in melanoma. The Skin Cutaneous Melanoma (TCGA) dataset was analysed for genetic alterations in PRELP. **Figure S5.** Association between PRELP expression, CD8^+^ T cell infiltration and activation as well as patients’ survival. **Figure S6.** A–C. Correlation of PRELP^high^ vs. PRELP^low^ samples with the expression of CCL5 and overall survival. **Table S1.** Primers used for qPCR analyses. **Table S2.** Datasets used and number of samples analyzed. **Table S3.** Correlation of PRELP expression with HLA class I and APM components using two different melanoma datasets. **Table S4.** Exploring the association between PRELP and immune infiltrates with the clinical outcome in SKCM datasets.

## Data Availability

All data generated or analyzed during this study are included either in this article or in the supplementary information files. The datasets generated during and/or analyzed during the current study are available from the corresponding author on reasonable request.

## Abbreviations

Ab: Antibody
APM: Antigen processing machinery
BM: Basement membrane
β_2_-m: β_2_-Microglobulin
CCL5: Chemokine ligand 5
CPTAC: Clinical Proteomic Tumor Analysis Consortium
CRC: Colorectal carcinoma
CTL: Cytotoxic T lymphocyte
CTLA4: Cytotoxic T lymphocyte antigen 4
ECM: Extracellular matrix
EMT: Epithelial mesenchymal transition
FCS: Fetal calf serum
FPKM: Fragments per kilo base per million mapped reads
GADPH: Glyceride aldehyd-3-phosphate dehydrogenase
GAG: Glycosaminoglycan
HC: Heavy chain
HCC: Hepatocellular carcinoma
HLA: Human leukocyte antigen
HR: Hazard ratio
HRP: Horse reddish peroxidase
ICP: Immune checkpoint
ICPi: Immune checkpoint inhibitor
IFN: Interferon
IRF: Interferon regulated factor
JAK: Janus kinase
LILR: Leukocyte immunoglobulin receptor
LMP: Low molecular weight protein
LRR: Leucine-rich repeat
luc: Luciferase
mAb: Monoclonal antibody
MFI: Mean fluorescence intensity
MHC: Major histocompatibility complex
MITF: Microphthalmia-associated transcription factor
NF-kB: Nuclear factor-kappa B
NK: Natural killer
OS: Overall survival
PD1: Programed death receptor 1
PD-L1: Programed death ligand 1
PG: Proteoglycan
PRELP: Purine-arginine-rich and leucine-rich protein
SKCM: Skin cutaneous melanoma
SLRP: Small leucine rich proteoglycan
STAT: Signal transducer and activator of transcription
TAP: Transporter associated with antigen processing
TAPBP: Tapasin
TCGA: The Cancer Genome Atlas
TGF-β: Transforming growth factor β
TIMER: Tumor Immune Estimation Resource
TME: Tumor microenvironment
TPM: Transcripts per kilo base million
