# Supplementary material for: Correlation of the tumor escape phenotype with loss of PRELP expression in melanoma
Source: J Transl Med. 2023 Sep 20;21:643. doi: 10.1186/s12967-023-04476-x (PMC10512569; doi:10.1186/s12967-023-04476-x)
Supplement: Supplementary file 1 — Additional file 1: Figure S1. PRELP expression in neoplastic and non-neoplastic human tissues and cells. Figure S2. Correlation plot of HLA-A and PRELP in 133 skin cancer cell lines (GENT2—skin cancer cell lines). Figure S3. Reconstitution of PRELP expression in melanoma cells. Overexpression of PRELP in melanoma cell lines was obtained after transfection of a PRELP expression vector in murine and human PRELPlow melanoma cells. Transfection with a mock vector served as control. Figure S4. Distribution of gene mutations of PRELP in melanoma. The Skin Cutaneous Melanoma (TCGA) dataset was analysed for genetic alterations in PRELP. Figure S5. Association between PRELP expression, CD8+ T cell infiltration and activation as well as patients’ survival. Figure S6. A–C. Correlation of PRELPhigh vs. PRELPlow samples with the expression of CCL5 and overall survival. Table S1. Primers used for qPCR analyses. Table S2. Datasets used and number of samples analyzed. Table S3. Correlation of PRELP expression with HLA class I and APM components using two different melanoma datasets. Table S4. Exploring the association between PRELP and immune infiltrates with the clinical outcome in SKCM datasets. [file 12967_2023_4476_MOESM1_ESM.pdf]

Additional file 1

Figure S1: PRELP expression in neoplastic and non-neoplastic human tissues and cells

A. Heterogeneous expression pattern of PRELP across 123 human healthy tissues and sub-tissues

The expression levels of PRELP given as TPM value were analysed using the Omic Horizon Expression database and presented as bar chart.

B. Reduced PRELP expression in melanoma cell lines (n=48) using qRT-PCR.

The PRELP expression of melanoma cell lines were compared to that of melanocytes. Using cycle of quantification/qualification (Cq) scale, the expression of melanoma cell lines were characterized for their PRELP expression.

C. PRELP of tumor skin cutaneous melanoma expression independent of tumor grade, tumor size, gender and age. The data are presented as transcript per million.

A

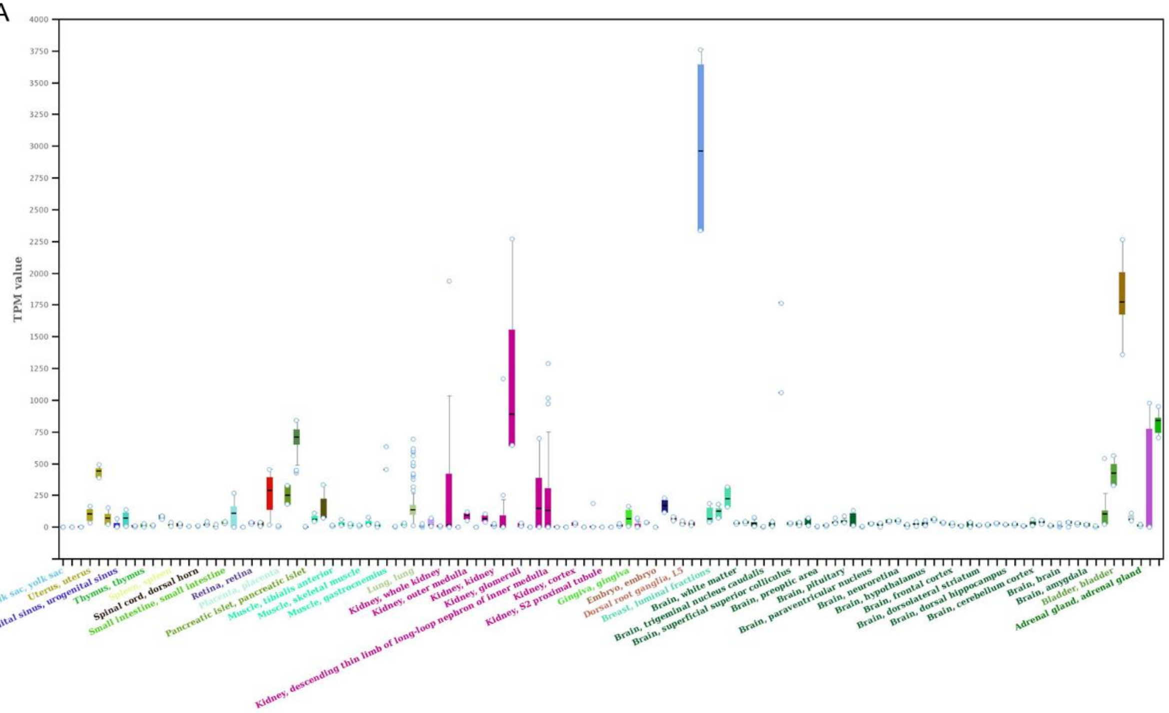

B

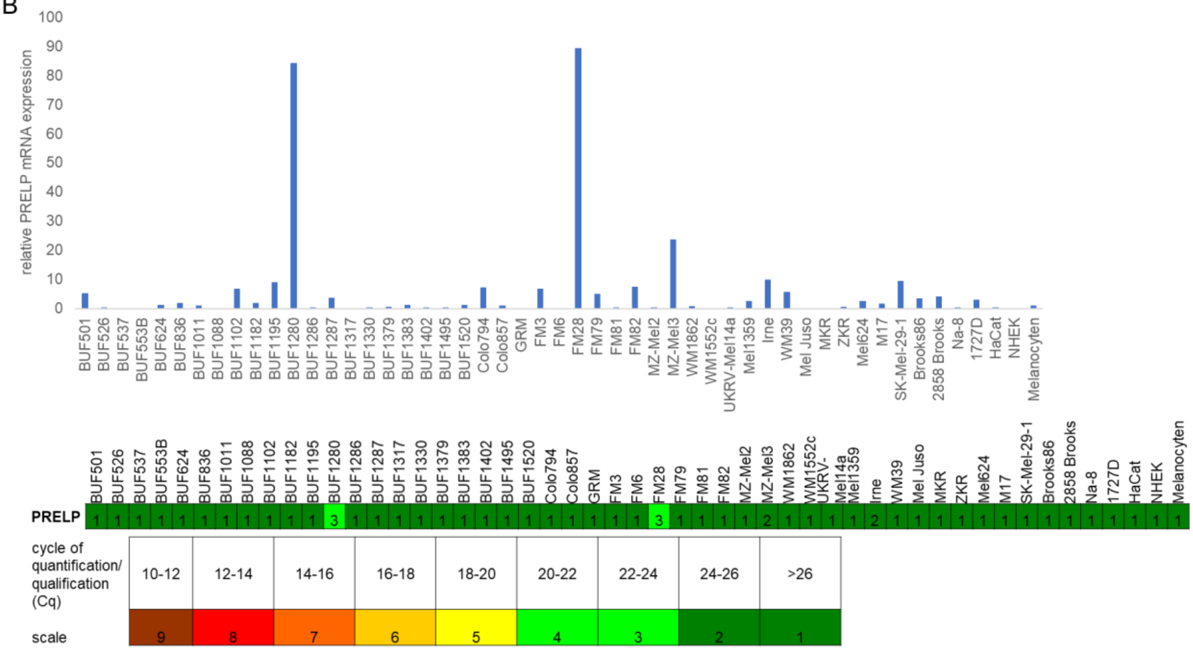
relative PRELP mRNA expression
100
90
80
70
60
50
40
30
20
10
0
BUF501
BUF526
BUF537
BUF553B
BUF624
BUF836
BUF1011
BUF1088
BUF1102
BUF1182
BUF1195
BUF1280
BUF1286
BUF1287
BUF1317
BUF1330
BUF1379
BUF1383
BUF1402
BUF1495
BUF1520
Colo794
Colo857
GRM
FM3
FM6
FM28
FM79
FM81
FM82
MZ-Mel2
MZ-Mel3
WM1862
WM1552c
UKRV-Mel14a
Mel1359
Irne
WM39
Mel Juso
MKR
ZKR
Mel624
M17
SK-Mel-29-1
Brooks86
2858 Brooks
Na-8
1727D
HaCat
NHEK
Melanocyten
PRELP
cycle of quantification/ qualification (Cq)
10-12
12-14
14-16
16-18
18-20
20-22
22-24
24-26
>26
scale
9
8
7
6
5
4
3
2
1

C

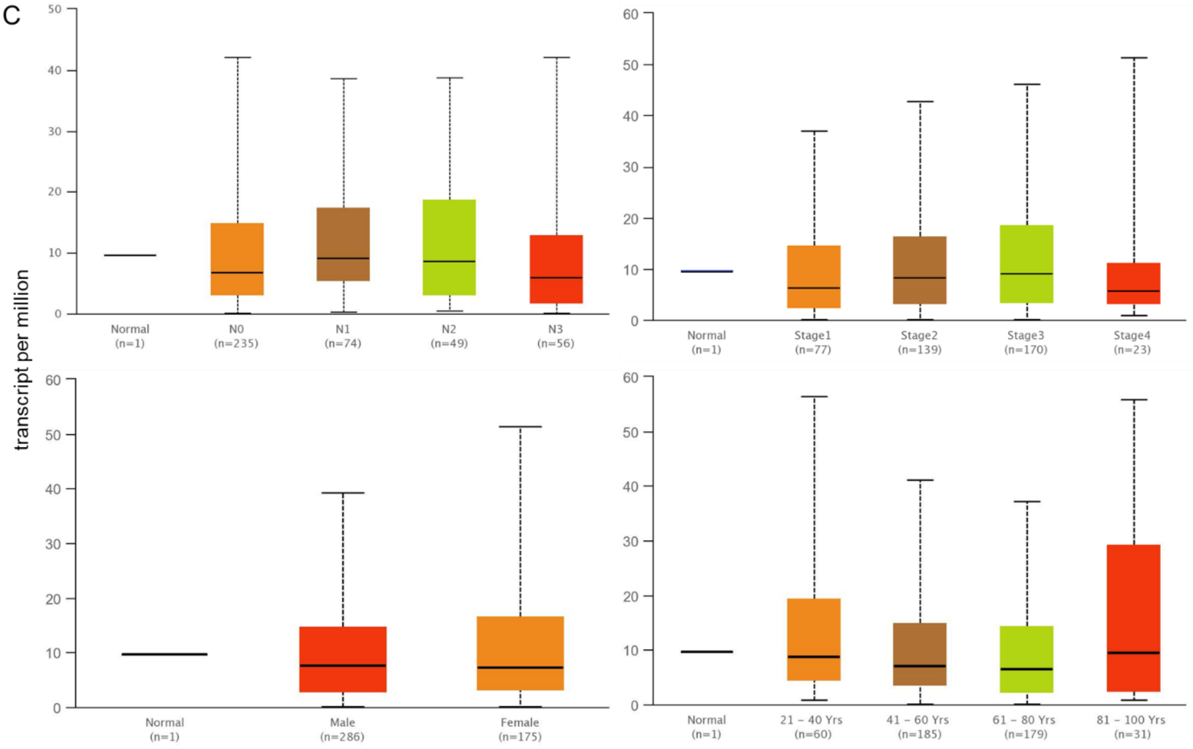
transcript per million
Normal (n=1)
N0 (n=235)
N1 (n=74)
N2 (n=49)
N3 (n=56)
Stage1 (n=77)
Stage2 (n=139)
Stage3 (n=170)
Stage4 (n=23)
Male (n=286)
Female (n=175)
21 - 40 Yrs (n=60)
41 - 60 Yrs (n=185)
61 - 80 Yrs (n=179)
81 - 100 Yrs (n=31)

Figure S2: Correlation plot of HLA-A and PRELP in 133 skin cancer cell lines (GENT2 – Skin cancer cell lines)

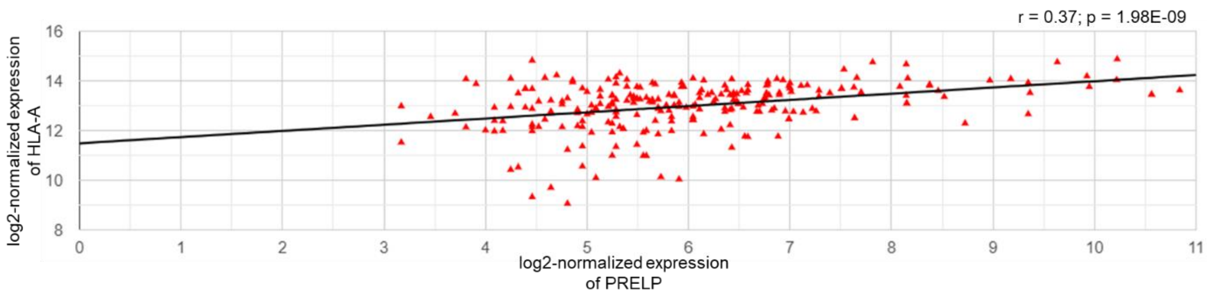

The PRELP expression levels in melanoma cells were correlated to HLA-A expression and presented as log2-normalized expression of HLA-A and PRELP.

Figure S3: Reconstitution of PRELP expression in melanoma cells. Overexpression of PRELP in melanoma cell lines was obtained after transfection of a PRELP expression vector in murine and human PRELP^{low} melanoma cells. Transfection with a mock vector served as control. PRELP mRNA expression was determined in PRELP^{low} B16F10 and Buf1088 cells and their PRELP transfectants as described in Material and Methods. qPCR data are shown as bar charts and represent the mean of at least three independent experiments.
The statistical significance is presented as * $p < 0.05$; *** $p < 0.001$.

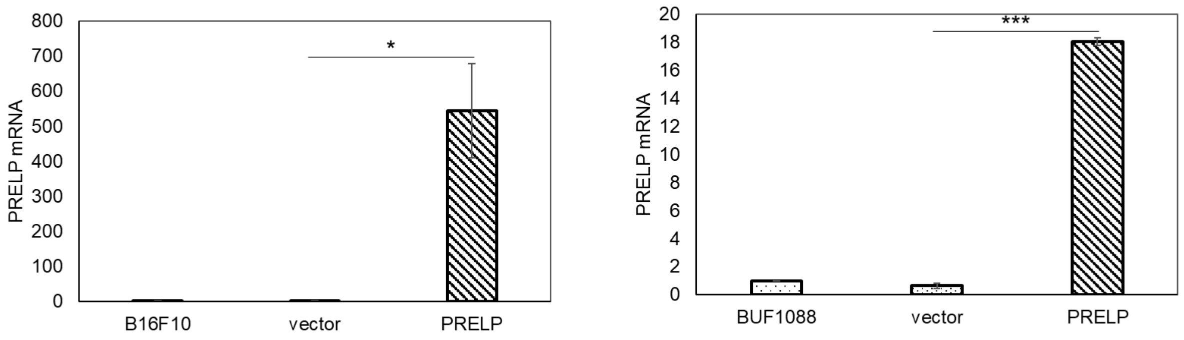

Figure S4: Distribution of gene mutations of PRELP in melanoma. The Skin Cutaneous Melanoma (TCGA) dataset was analysed for genetic alterations in PRELP.

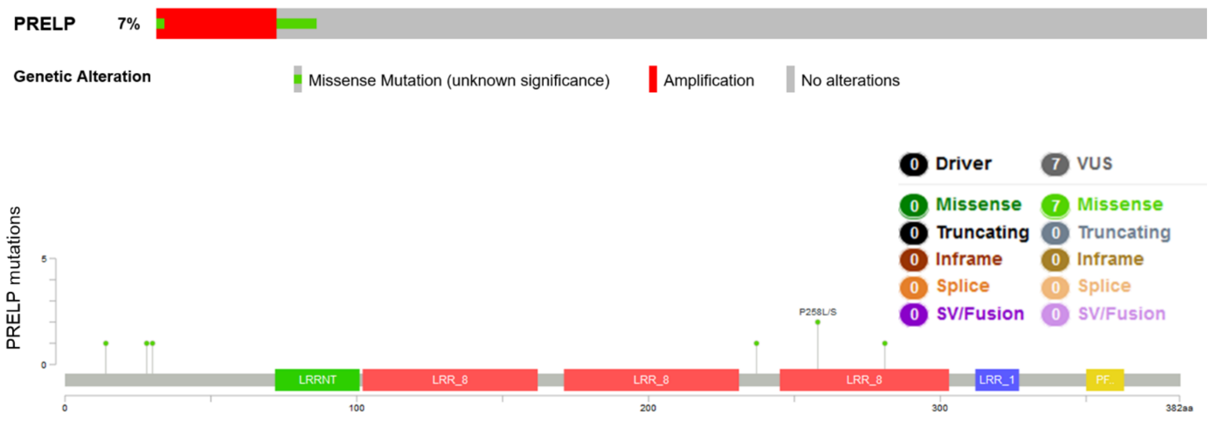

Figure S5: Association between PRELP expression, CD8^{+} T cell infiltration and activation as well as patients' survival

A. Association between infiltration levels of CD8^{+} T cell subsets and the cumulative overall survival of tumor skin cutaneous melanoma and metastatic melanoma datasets with different PRELP expression levels. In tumor skin cutaneous melanoma, the prognostic signature was built on the immune infiltration of naive, central memory and effector memory CD8^{+} T cells and the expression of PRELP profiled by xCell algorithm.

B. In metastatic melanoma, the prognostic signature built by the immune infiltration of naive, central memory and effector memory CD8^{+} T cells and the expression of PRELP profiled by xCell algorithm.

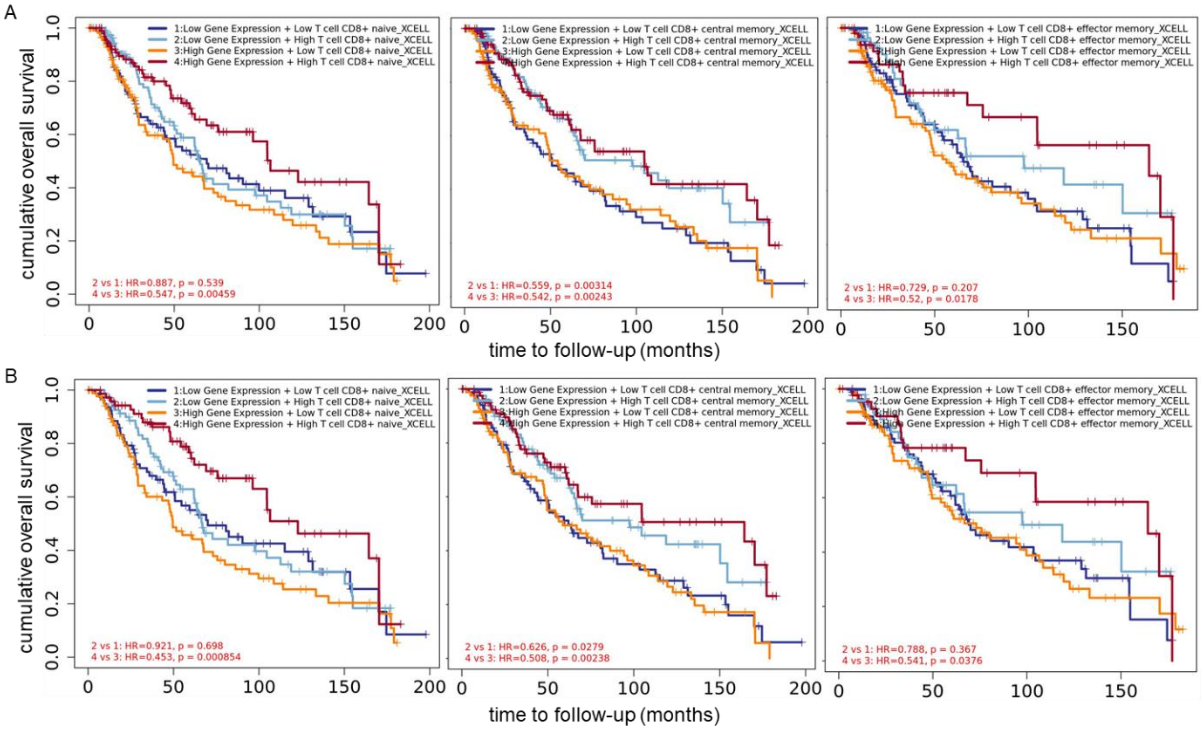

Figure S6:

A, B, C. Correlation of PRELP^{high} vs. PRELP^{low} samples with the expression of CCL5 and overall survival

The TCGA dataset (Tumor Skin Cutaneous Melanoma – TCGA) was analyzed regarding patients' overall survival using the Kaplan-Meier curve for CCL5 expression independent of PRELP expression (A), PRELP^{high} (B) and PRELP^{low} (C) samples.

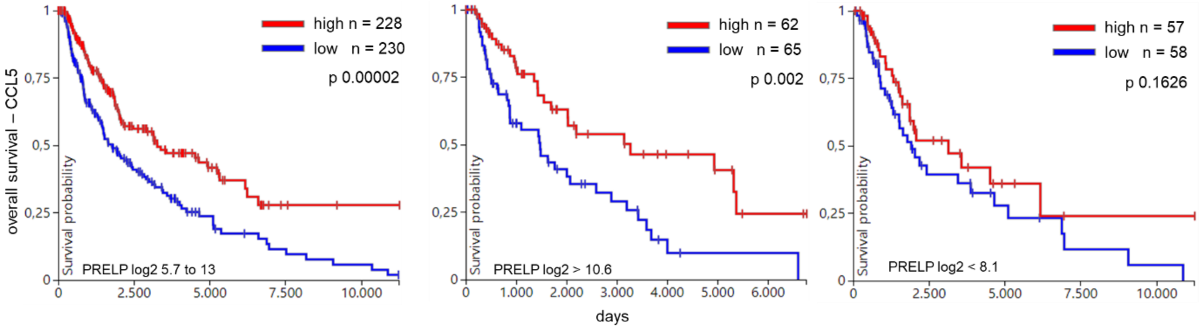

Table S1: Primers used for qPCR analyses.

**murine**		
**gene**	**Forward**	**reverse**
PRELP	ACTGTCCACGGGAATGCTAC	CGTGGCATTCTGGAAAGATT
H-2Lq	ACACGATCCAGCGGATGT	CAGGTAGGCCTTGTAATGCTCT
TAP1	TGCCTAAGAAGCTGGGAAAA	GTAAGCCAAGGCCTCCTTCT
TAP2	CGGTGCTAAAGGAGATCCAG	CCATCACCCTCCGTATGACT
TAPBP	ACACTGCGAGATGAGCCGCTTC	TGAGGACGGTCAGCACCACTGT
PSMB9	TCTTCTGTGCCCTCTCAGGT	TGGTCCCAGCCAGCTACTAT
PSMB8	CGGGACAGATGTTTTCCACT	CACTTTCACCCAACCGTCTT
PSMB10	ACCCACATGGTTCCTACAGC	GTGATCACACAGGCATCCAC
B2M	GCACGCAGAAAGAAATAGCA	ACAGAGGGTTTGGCATATGAT
NLRC5	TGGAGGAGGTCAGTTTGC	ATGCTCCTGATTGCTGTGTAG
IRF1	ACTCGAATGCGGATGAGACC	TGCTTTGTATCGGCCTGTGT
ß-actin	TCTGCTGGAAGGTGGACAGT	CCTCTATGCCAACACAGTGC
GAPDH	AAGGTCATCCCAGAGCTGAA	CTGCTTCACCACCTTCTTGA
**human**		
**gene**	**Forward**	**reverse**
PRELP	TCCCAATCTTGCCTTCATTC	GTCATGGAACGCCACTAGGT
HLA-ABC	GCCTACCACGGCAAGGATTAC	GGTGGCCTCATGGTCAGAGA
TAP1	GGAATCTCTGGCAAAGTCCA	TGGGTGAACTGCATCTGGTA
TAP2	CCAAGACGTCTCCTTTGCAT	TTCATCCAGCAGCACCTGTC
TAPBP	TGGGTAAGGGACATCTGCTC	ACCTGTCCTTGCAGGTATGG
PSMB9	TGCTGCATCCACATAACCAT	TGTGCACTCTCTGGTTCAGC
PSMB8	TCTGCGTCATCAGCAAGAAC	GCCATTCAGGAAGTGTCCAT
PSMB10	GGGCTTCTCCTTCGAGAACT	CAGCCCCACAGCAGTAGATT
B2M	CTCGCGCTACTCTCTCTT	AAGACCAGTCCTTGCTGA
NLRC5	CAATTTGATGAGGAGGGCAC	GTGAGTAAGCAAGGCCAAGG
IRF1	GCAGCTACACAGTTCCAGG	GTCCTCAGGTAATTTCCCTTCCT
ß-actin	ACTCTTCCAGCCTTCCTTCC	AGCACTGTGTTGGCGTACAG
GAPDH	CTGGTAAAGTGGATATTGTTGCCAT	TGGAATCATATTGGAACATGTAAACC

Table S2: Datasets used and number of samples analyzed.

	Datasets	no. of samples	reference
1	GENT2 - U133Plus2	~ 44,000	887 GEO datasets (51, 52)
2	GENT2 - U133A	~ 23,000	358 GEO datasets (51, 52)
3	GENT2 – skin cancer cell lines	247 cancer; 32 normal tissues	(51, 52)
4	HCMDB - EXP00026	16 healthy tissues; 46 primary tumors	GEO ID: GSE15605 (82)
5	HCMDB - EXP00365	40 metastases; 16 primary tumors	GEO ID: GSE7553 (63)
6	HCMDB - EXP00367	4 healthy; 26 primary tumors	GEO ID: GSE7553 (63)
7	mixed melanoma (metastasis)	83	GEO ID: GSE8401 (83)
8	mixed melanoma	7186	GEO ID: GSE115978 (84)
9	tumor skin cutaneous melanoma – TCGA	480	(85)
10	total $CD8^+$ T cells from healthy human donors	15	(86)

Table S3: Correlation of PRELP expression with HLA class I and APM components using two different melanoma datasets.

**PRELP**	**mixed melanoma (metastasis)**		**Tumor Skin Cutaneous Melanoma – TCGA**	
	**83 samples**		**470 samples**	
	**r value**	**p value**	**r value**	**p value**
HLA-A	0.342	1.55E-03	0.12	9.13E-03
HLA-B	0.357	9.13E-04	0.068	0.139
HLA-C	0.37	5.81E-04	0.084	0.069
TAP1	0.07	0.531	0.018	0.692
TAP2	-0.062	0.579	-0.073	0.113
TAPBP	0.256	0.02	0.211	3.82E-06
CALR	-0.085	0.444	-0.015	0.738
CANX	-0.381	3.82E-04	-0.045	0.328
LMP2	-0.04	0.722	-0.004	0.928
LMP7	0.128	0.249	0.028	0.541
LMP10	0.157	0.155	0.109	0.018

Table S4: Exploring the association between PRELP and immune infiltrates with the clinical outcome in SKCM datasets.

The multivariable Cox proportional hazard model was generated by using XCELL algorithm in the TIMER database. Each cell type of the table corresponds to an independent Cox model. The hazard ratio (HR) and p value for the Cox model were displayed.

**immune infiltrates**	**HR**	**p-value**	**clinical relevance**
T cell $CD8^+$			
SKCM (n=471)	-4.079	1.25E-04	decreased risk
SKCM-Metastasis (n=368)	-3.758	6.19E-04	decreased risk
T cell $CD4^+$			
SKCM (n=471)	-0.899	4.79E-01	not significant
SKCM-Metastasis (n=368)	-0.612	7.69E-01	not significant
Tregs			
SKCM (n=471)	-1.044	4.21E-01	not significant
SKCM-Metastasis (n=368)	-0.5	8.20E-01	not significant
B cell			
SKCM (n=471)	-2.533	2.81E-02	decreased risk
SKCM-Metastasis (n=368)	-1.995	1.25E-01	not significant
Neutrophil			
SKCM (n=471)	1.212	3.58E-01	not significant
SKCM-Metastasis (n=368)	0.567	7.95E-01	not significant
Monocyte			
SKCM (n=471)	-3.077	5.87E-03	decreased risk
SKCM-Metastasis (n=368)	-2.832	1.59E-02	decreased risk
Macrophage			
SKCM (n=471)	-3.934	3.01E-04	decreased risk
SKCM-Metastasis (n=368)	-3.46	2.23E-03	decreased risk
myeloid dendritic cell			
SKCM (n=471)	-2.619	2.30E-02	decreased risk
SKCM-Metastasis (n=368)	-2.375	5.47E-02	decreased risk
NK cell			
SKCM (n=471)	-1.206	3.50E-01	not significant
SKCM-Metastasis (n=368)	-1.232	4.32E-01	not significant
mast cell			

SKCM (n=471)	0.916	4.74E-01	not significant
SKCM-Metastasis (n=368)	1.144	4.82E-01	not significant
cancer associated fibroblast			
SKCM (n=471)	-0.544	6.20E-01	not significant
SKCM-Metastasis (n=368)	-0.268	8.96E-01	not significant
common lymphoid progenitor			
SKCM (n=471)	-1.661	1.87E-01	not significant
SKCM-Metastasis (n=368)	-2.036	1.19E-01	not significant
common myeloid progenitor			
SKCM (n=471)	2.284	4.84E-02	increased risk
SKCM-Metastasis (n=368)	2.027	1.12E-01	not significant
endothelial cell			
SKCM (n=471)	0.055	7.21E-01	not significant
SKCM-Metastasis (n=368)	0.099	9.25E-01	not significant
Eosinophil			
SKCM (n=471)	1.866	1.21E-01	not significant
SKCM-Metastasis (n=368)	0.834	6.54E-01	not significant
granulocyte-monocyte progenitor			
SKCM (n=471)	-0.516	6.25E-01	not significant
SKCM-Metastasis (n=368)	-0.642	7.24E-01	not significant
hematopoietic stem cell			
SKCM (n=471)	-0.906	4.74E-01	not significant
SKCM-Metastasis (n=368)	-0.897	6.18E-01	not significant
gamma delta T cell			
SKCM (n=471)	-1.884	1.18E-01	not significant
SKCM-Metastasis (n=368)	-1.656	2.29E-01	not significant
NK T cell			
SKCM (n=471)	1.342	2.96E-01	not significant
SKCM-Metastasis (n=368)	1.895	1.55E-01	not significant
